# Chaotic attractor hopping yields logic operations

**DOI:** 10.1371/journal.pone.0209037

**Published:** 2018-12-21

**Authors:** K. Murali, Sudeshna Sinha, Vivek Kohar, Behnam Kia, William L. Ditto

**Affiliations:** 1 Department of Physics, Anna University, Chennai, India; 2 Department of Physical Sciences, Indian Institute of Science Education and Research Mohali, Knowledge City, SAS Nagar, Sector 81, Manauli, Punjab, India; 3 Nonlinear Artificial Intelligence Lab, Department of Physics, North Carolina State University, Raleigh, NC, United States of America; 4 The Jackson Laboratory, Bar Harbor, ME, United States of America; University of the West of England, UNITED KINGDOM

## Abstract

Certain nonlinear systems can switch between dynamical attractors occupying different regions of phase space, under variation of parameters or initial states. In this work we exploit this feature to obtain reliable logic operations. With logic output 0/1 mapped to dynamical attractors bounded in distinct regions of phase space, and logic inputs encoded by a very small bias parameter, we explicitly demonstrate that the system hops consistently in response to an external input stream, operating effectively as a reliable logic gate. This system offers the advantage that very low-amplitude inputs yield highly amplified outputs. Additionally, different dynamical variables in the system yield complementary logic operations in parallel. Further, we show that in certain parameter regions noise aids the reliability of logic operations, and is actually necessary for obtaining consistent outputs. This leads us to a generalization of the concept of Logical Stochastic Resonance to attractors more complex than fixed point states, such as periodic or chaotic attractors. Lastly, the results are verified in electronic circuit experiments, demonstrating the robustness of the phenomena. So we have combined the research directions of Chaos Computing and Logical Stochastic Resonance here, and this approach has potential to be realized in wide-ranging systems.

## Introduction

Nonlinear systems yield a rich gamut of dynamical behaviors that range from fixed points and limit cycles of varying periodicities, to chaotic attractors. In this work we will exploit the presence of dynamical attractors localized in different regions of phase space, and the possibility of hopping between such attractors, to obtain logic operations.

Consider a general nonlinear system of the form:

$$\begin{array}{ccc} \dot{x} & = & y-g(x),\\ \dot{y} & = & -ay-x+b+I+f(t), \end{array}$$ (1)

where *f*(*t*) is a periodic forcing signal, *g*(*x*) is a nonlinear function, *b* is a constant bias and *I* is an input signal. Specifically, consider a simple easily implementable piecewise linear form for

$$\begin{array}{c} g\left(x\right)=c_{1}x+\frac{1}{2}\left(c_{2}-c_{1}\right)\left(\right|\left(x+1\right)\left|-\right|\left(x-1\right)\left|\right) \end{array}$$ (2)

and *f*(*t*) = *A* sin(*ωt*), where *ω* is the frequency and *A* is the amplitude of the periodic forcing. These dimensionless coupled first order differential equations underlie the readily implementable MLC circuit [1].

**Fig 1:**
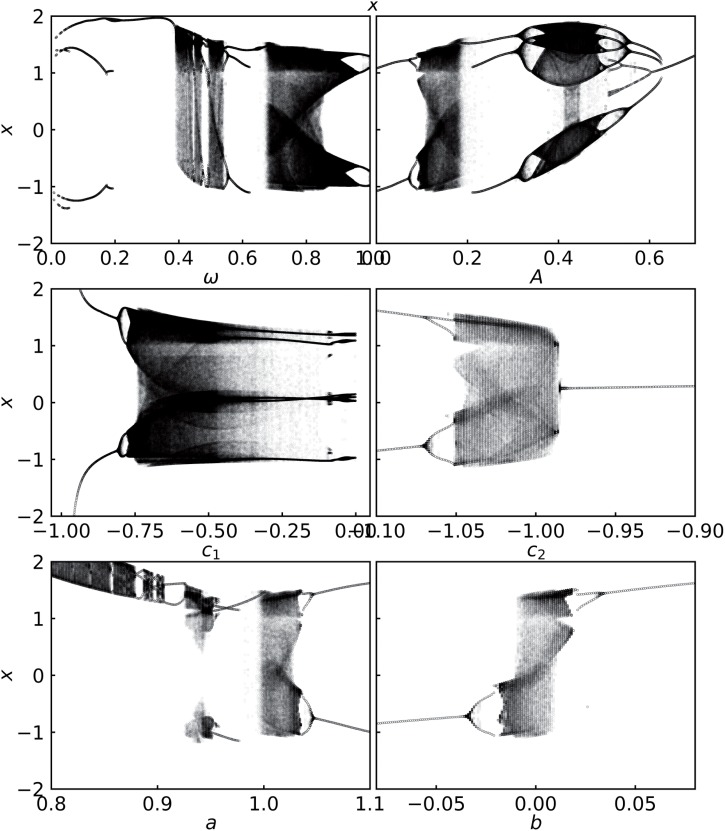
Bifurcation diagrams displaying the distinct dynamical attractors obtained with respect to the different parameters in the system (cf. Eqs 1 and 2). Frequency *ω*, amplitude *A*, *c*_1_, *c*_2_, *a* and bias parameter *b*. While one of the parameters is being varied, the other parameters (whichever are relevant) are fixed at: *a* = 1.015, *c*1 = −0.55, *c*2 = −1.02, *ω* = 0.74, *A* = 0.11, *b* = 0 in Eq 1.

The bifurcation diagrams of the system with respect to all the different parameters are shown in Fig 1, depicting the richness of behaviors which may be exploited for implementing different logic operations. Specifically we seek attractors in parameter space that occupy clearly distinct regions. The most suitable parameter that offers this feature, as well as the simplest one to manipulate, is the bias parameter *b*. So in this work we will use the patterns evident in the bifurcation diagram of the system, with respect to bias *b*, to design logic gates.

In order to conceive of a mapping of the dynamics to logic operations, we need to specify the inputs-to-output correspondence. We first focus on the encoding of logic inputs. In general, *N* logic inputs are encoded by *N* square waves which constitute the input signal *I* in Eq 1. In particular, for two logic inputs, the input signal *I* is the sum *I*_1_ + *I*_2_, with *I*_1_ and *I*_2_ encoding the two logic inputs. Since the logic inputs can be either 0 or 1, they can combine to give four logic input sets (*I*_1_, *I*_2_): (0, 0), (0, 1), (1, 0) and (1, 1), with the input sets (0, 1) and (1, 0) giving rise to the same *I*. This implies that the four input conditions (*I*_1_, *I*_2_) reduce to three distinct values of *I*. Hence, the input signal *I*, generated by adding two independent uncorrelated input signals, is a 3-level aperiodic waveform. In this work the input signal *I* will be considered to be of *very low amplitude*, compared to the typical size of the chaotic attractor. The central idea here rests on the capability of the nonlinear system to yield a large response, such as a very different dynamical attractor, in response to a very small input signal.

Now, this nonlinear system is capable of exhibiting attractors that are bounded in different regions of phase space. For instance, it can give rise to attractors where the value of the *x* (or *y*) variable is entirely positive, as well as attractors whose *x* (or *y*) values are entirely negative, under variation of the small input signal *I*. Dynamically, these attractors may be fixed points, periodic cycles or even chaotic attractors. So as the value of *I* switches, i.e. the input set switches, we observe that the attractors can jump from a certain sector of phase space to a very different sector. This is the feature which we will exploit to implement a robust input-output correspondence in this system [2–11].

So the dynamical attractor of the system will yield the logic output. For instance, if *x*(*t*) (or *y*(*t*)) is greater than *x*_*thresh*_ or *y*_*thresh*_) respectively, it is mapped to logic output 1, and if *x*(*t*) (or *y*(*t*)) is lower than *x*_*thresh*_ or *y*_*thresh*_) respectively, it is mapped to logic output 0. The thresholds for output determination *x*_*thresh*_ and *y*_*thresh*_ can be suitably chosen, and are typically close to zero. As mentioned earlier, specifically, we can have *x*_*thresh*_ = *y*_*thresh*_ = 0, namely we can consider the output to be a logical 1 if the inputs yield a positive attractor, and the output to be a logical 0 if it is a negative attractor, i.e. if *x*(or *y*) < 0, Logic Output is 0 and if *x*(or *y*) > 0, Logic Output is 1.

## Results

**Fig 2:**
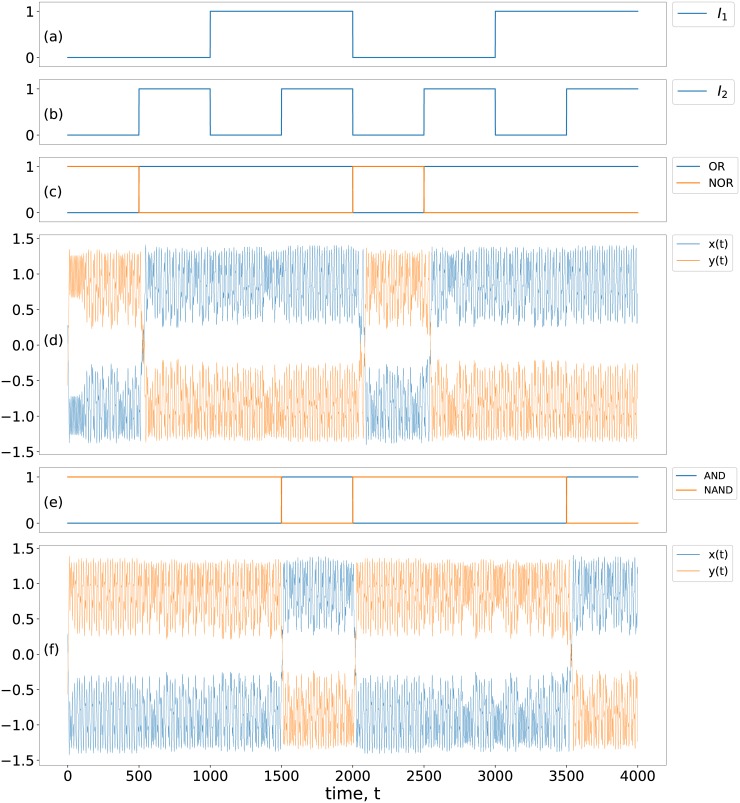
**Logic output from attractor hopping**: Panels (a) and (b) show a stream of inputs *I*_1_ and *I*_2_. Panel (c) shows the expected OR/NOR logic output and (e) shows the AND/NAND logic output corresponding to logic input set (*I*_1_, *I*_2_). Panels (d) and (f) show outputs *x*(*t*) and *y*(*t*) of the nonlinear system given by Eq 1 with *a* = 1.015, *c*_1_ = −0.55, *c*_2_ = −1.02, *A* = 0.11, *ω* = 0.74. The input signals take value −0.002 when logic input is 0 and value 0.002 when logic input is 1, and logic output is 1 when *x*(*t*) (or *y*(*t*)) > 0, and logic output is 0 when *x*(*t*) (or *y*(*t*)) < 0. In (d), bias *b* = −0.002, and clearly the *x* variable yields a consistent logical OR output, while the *y* variable yields a consistent NOR logic output. In (f), bias *b* = +0.002, and clearly the *x* variable yields a consistent logical AND output, while the *y* variable yields the complementary NAND logic gate.

**Fig 3:**
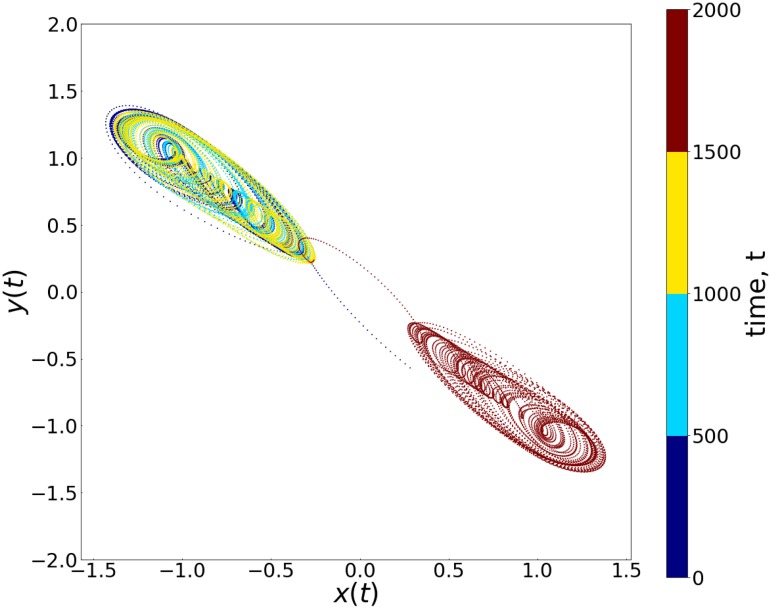
Phase portraits of the dynamical attractors arising from the different input sets in Fig 2 (with *a* = 1.015, *c*_1_ = −0.55, *c*_2_ = −1.02, *A* = 0.11, *ω* = 0.74 in Eq 1). Here the time evolution under different input sets is depicted in different colors. It is clearly evident that the trajectory switches between chaotic attractors in two distinct quadrants as the logic input sets change, yielding appropriate output states.

We will now demonstrate here that a given set of inputs (*I*_1_, *I*_2_) yields an output in accordance with the truth tables of the basic logic operations shown in Table 1. Crucially, the different outputs will arise from the *chaotic attractor hopping induced by the input stream*. We present explicit examples of this phenomenon, from numerical simulations, in Figs 2 and 3. These figures show illustrative cases of positive and negative chaotic attractors yielding Logic Output 1 and 0 respectively, under a stream of external input signals. So as the system receives different inputs it switches between these qualitatively different dynamical attractors, yielding the appropriate output. Fig 3 specifically shows the two one-band/single-scroll chaotic attractors that occupy distinct regions of phase space, characterizing the two outputs. So, as the system hops between these chaotic attractors, the output toggles between 0 and 1. Significantly, the very low-amplitude input signals yield highly amplified outputs. For instance, in our representative example, the input signal *I* = 0.002 results in dynamical attractors that differ on average by ∼2, i.e. approximately two orders of magnitude larger. Namely, the *input signal (which is of the order of* 10^−3^*) is very small, vis-a-vis the size of chaotic attractor (which is of the order of* 1*)*, implying that a small change in the system yields a significantly different dynamical outcome. Also note that the response time of the system is very short, with the system taking of the order of microseconds on average to switch between the desired states, leading to low latency.

**Table 1:** Relationship between the two inputs and the output of the fundamental OR, AND, NOR and NAND operations. Note that the four distinct possible input sets (0, 0), (0, 1), (1, 0) and (1, 1) reduce to three conditions as (0, 1) and (1, 0) are symmetric. Note that *any* logical circuit can be constructed by combining the fundamental NOR (or the NAND) gates [12].

Input Set (*I*_1_, *I*_2_)	OR	AND	NOR	NAND
(0,0)	0	0	1	1
(0,1)/(1,0)	1	0	0	1
(1,1)	1	1	0	0

Further, under a different bias parameter *b* in Fig 2(f), we also obtain a consistent OR logic operation, again by switching between chaotic attractors confined in distinct quadrants of phase space. So the system has the capablity of implementing different logic operations *flexibly* through a simple change of bias parameter, leading to potentially reconfigurable logic gates.

Additionally, the *x* and *y* variables yield *complementary* logic outputs in parallel. So in the specific examples presented in Fig 2d and 2f, variable *x* yields a consistent AND/OR gate response, while variable *y* yields a consistent NAND/NOR gate response. Thus this two-dimensional system allows us to *implement operations in parallel* by simultaneously yielding the two complementary logic outputs.

### Quantifying the reliability of obtaining a logic output

We can quantify the consistency (or reliability) of obtaining a given logic output by calculating the probability, denoted by *P*(*logic*), of obtaining the desired logic output for different input sets. *P*(*logic*) is estimated from numerical simulations by calculating the ratio of the number of successful runs to the total number of runs. Each run consists of a permutation of the inputs sets (0, 0), (0, 1)/(1, 0), (1, 1) fed sequentially to the system. If the logic output obtained from the system is the desired output for *all* input sets in the run, it is considered a “success”. Even if one input set returns a wrong output, the full run is considered a “failure”. So this quantity offers a very stringent measure of reliability. When *P*(*logic*) is 1, the logic operation is very reliable, as the system yields the correct output in response to all the input sets (*I*_1_, *I*_2_) provided to it. Specifically in the numercial results presented here we sample 10^3^ runs.

**Fig 4:**
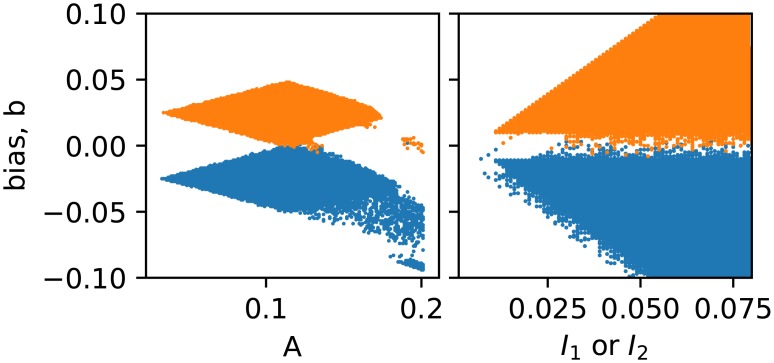
Regions in the parameter space of bias *b* (*y*-axis) and forcing amplitude *A* (*x*-axis, left), input *I*_1_/*I*_2_ (*x*-axis, right), and where the probability of obtaining NAND (orange) and NOR (blue) logic is 1. Here *a* = 1.015, *c*_1_ = −0.55, *c*_2_ = −1.02, *ω* = 0.75 and the inputs *I*_1_/*I*_2_ take values −0.025/0.025 for logic input 0/1 in (a) and *A* = 0.14 in (b). Here the dynamical attractors may be limit cycles or chaotic attractors.

It is evident from Fig 4, which shows *P*(*logic*) obtained from numerical simulations, that the fundamental logic operations NOR and NAND are realized consistently in an optimal band of moderate forcing amplitude *A* and bias *b*. The logic response is 100% accurate in this reasonably wide window. That is, in a broad region of parameter space the system yields outputs, in response to external inputs, in complete accordance with the fundamental logic functionalities shown in the truth tables (cf. Table 1). Also, importantly, a simple switch in bias *b* changes the logic gate from NOR/OR to NAND/AND. This implies that the system can operate flexibly as a NOR/OR logic gate or a NAND/AND logic gate, with the small bias parameter having the capacity to morph the system to operate as different logic gates.

### Experimental verification

**Fig 5:**
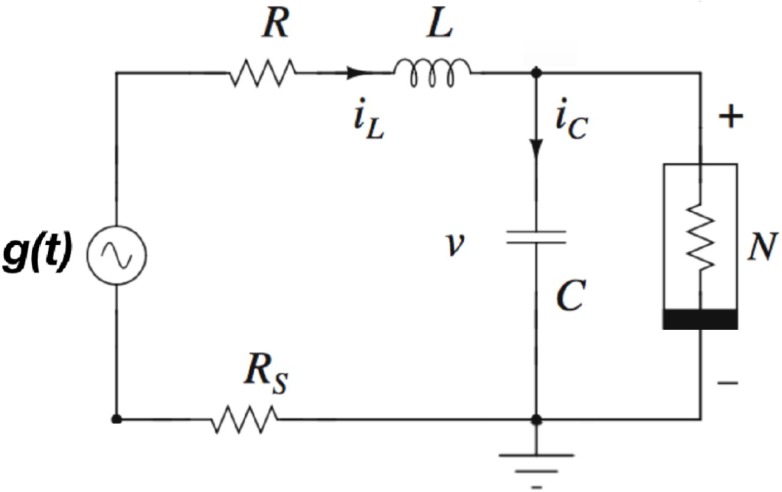
Schematic of a simple electronic circuit, known as the MLC circuit [1], implementing the rescaled dynamical equations given in Eq 1. In the circuit, the voltage across the capacitor *C*, the current through the inductor *L* and the external forcing signal *g*(*t*) correspond to *x*, *y* and *b* + *I* + *f*(*t*) in Eq 1 respectively. The nonlinear element *N* is the Chua’s diode implemented as in [1], with rescaled piecewise-linear characteristic curve $g\left(x\right)=c_{1}x+\frac{1}{2}\left(c_{2}-c_{1}\right)\left(\right|\left(x+1\right)\left|-\right|\left(x-1\right)\left|\right)$.

**Fig 6:**
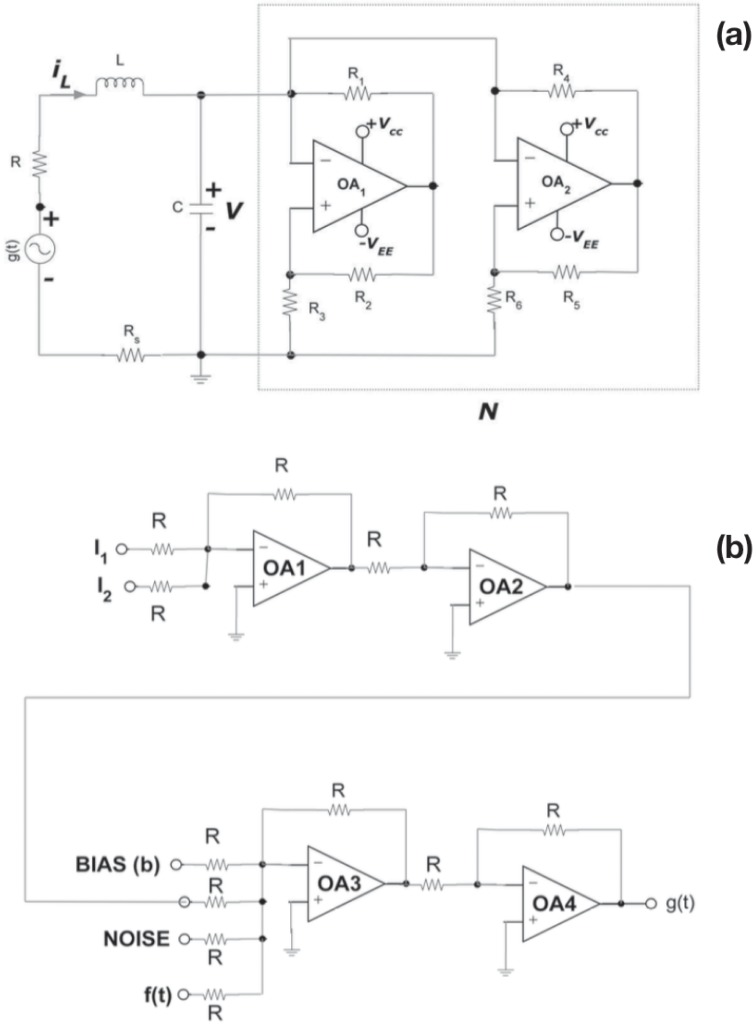
(a): Realization of MLC circuit using two op-amps (AD712, TL082, AD844, or equivalent) and six linear resistors to implement the Chua’s diode (*N*). The resistors *R* = 1340 Ω, *R*_1_ = *R*_2_ = 22 *k*Ω, *R*_3_ = 3.3 *k*Ω, *R*_4_ = *R*_5_ = 220 Ω, *R*_6_ = 2.2 *k*Ω and *R*_*s*_ = 20 Ω. The capacitor *C* = 10 *nF* and the inductor *L* = 18 *mH* (TOKO type 10RB or equivalent). Here *g*(*t*) is the input driving signal, the capacitor voltage is *V*(*t*) and the inductor current is *i*_*L*_. The current *i*_*L*_ is sensed through the resistor *R*_*s*_ to give the voltage *V*_*L*_ [1]. (b) Circuit for generating the driving signal *g*(*t*). Here op-amps OA1—OA4 are realized with AD712. All the resistors are fixed as *R* = 10 *k*Ω. The power-supply to op-amps and the bias voltage (*b*) are drawn from Agilent or Keysight E3631A DC Power Supply. The sinusoidal signal *f*(*t*) and the noise signal are drawn from Agilent or Keysight 33522A, Function/Arbitrary Waveform Generator.

Now we will verify this concept in electronic circuit analogs of the nonlinear system described by Eq 1, and ascertain its robustness in experiments. The schematic of the circuit realization of the simple non-autonomous MLC circuit is shown in Fig 5. It contains a capacitor, an inductor, a linear resistor, an external periodic forcing *g*(*t*) and only one nonlinear element, namely, the Chua’s diode (*N*) [1]. The complete circuit implementation of MLC circuit is depicted in Fig 6(a). To measure the inductor current *i*_*L*_ in our experiments, we insert a current sensing resistor *R*_*s*_ as shown in Fig 6a to give the voltage *V*_*L*_ [1]. By applying Kirchhoff’s laws to this circuit, the governing equations for the voltage (*V*) across the capacitor *C* and the current *i*_*L*_ through the inductor *L* are represented by the rescaled Eq 1 [1]. Two op-amps (AD712, TL082, AD844, or equivalent) and six linear resistors are used to implement the Chua’s diode (*N*). The parameters of the circuit elements are fixed as resistors *R* = 1340 Ω, *R*_1_ = *R*_2_ = 22 *k*Ω, *R*_3_ = 3.3 *k*Ω, *R*_4_ = *R*_5_ = 220 Ω, *R*_6_ = 2.2 *k*Ω and *R*_*s*_ = 20 Ω. The capacitor *C* = 10 *nF* and the inductor *L* = 18 *mH* (TOKO type 10RB or equivalent). The frequency of the external sinusoidal force *f*(*t*) as in Fig 6(b) is fixed at 8890 *Hz*. The circuit of Fig 6(b) is used to generate the driving signal *g*(*t*) for the circuit of Fig 6(a). In the circuit of Fig 6(b), *g*(*t*) is basically generated by a set of op-amp summing amplifiers by adding the logic input signals *I*_1_ and *I*_2_, external bias voltage (*b*), external noise signal and the sinusoidal signal *f*(*t*). All the op-amps are employed with AD712 (or TL082 or AD844 or equivalent). The voltage supply for all the op-amps are fixed at ±9 *V*. All the resistors are fixed as *R* = 10 *k*Ω.

**Fig 7:**
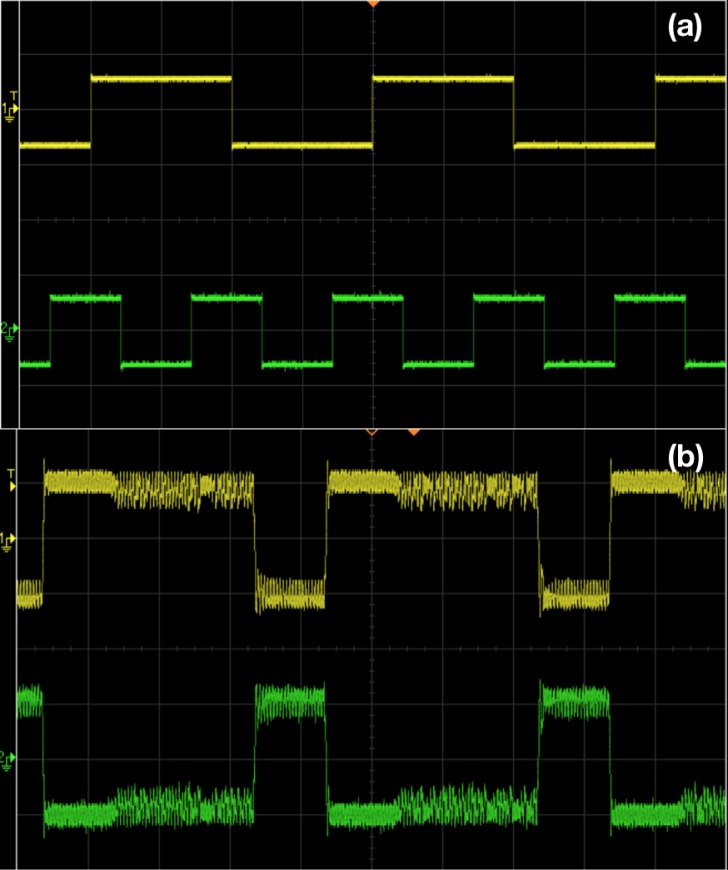
Realization of OR/NOR logic gates through chaotic attractor hopping in electronic circuit experiments. Panel (a) shows stream of inputs *I*_1_ and *I*_2_ (which take value −10 *mV* when logic input is 0 and value 10 *mV* when logic input is 1). Panel (b) shows the voltage *V*(*t*) (yellow) clearly indicating a logical OR output (with *V*(*t*) > 0 being logic output 1, and *V*(*t*) < 0 being logic output 0). The output voltage *V*_*L*_ (green) yields the complementary NOR logic gate response. The amplitude *A* of the sinusoidal signal is 150 *mV* and frequency is 8890 *Hz*. The bias voltage *b*, is fixed as 10 *mV*. For panel (a), the scale in the traces are: 20 *mV*/Div (*Y*-axis) and 5 *mS*/Div (*X*- axis). For panel (b), the scale in the traces are: 100 *mV*/Div (*Y*-axis) and 5 *mS*/Div (*X*- axis). The oscilloscope used is Agilent or Keysight DSOX2012A.

**Fig 8:**
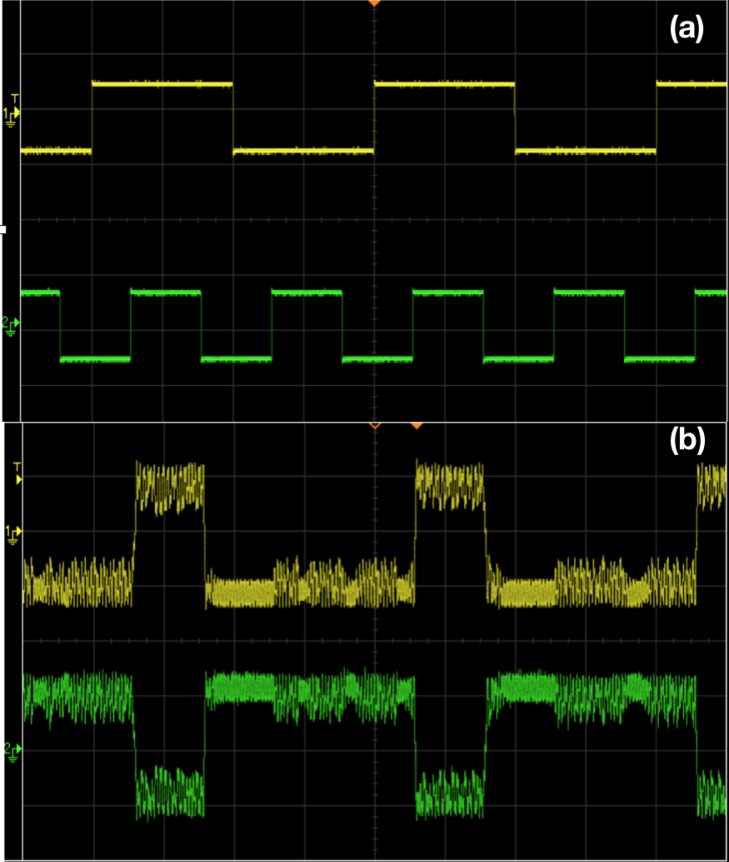
Realization of AND/NAND logic gates through chaotic attractor hopping in electronic circuit experiments. Panel (a) shows stream of inputs *I*_1_ and *I*_2_ (which take value −10 *mV* when logic input is 0 and value 10 *mV* when logic input is 1). Panel (b) shows the voltage *V*(*t*) (yellow) clearly indicating a logical AND output (with *V*(*t*) > 0 being logic output 1, and *V*(*t*) < 0 being logic output 0). The output voltage *V*_*L*_ (green) yields the complementary NAND logic gate response. The amplitude *A* of the sinusoidal signal is 150*mV* and frequency is 8890 *Hz*. The bias voltage *b*, is fixed as −10 *mV*. For panel (a), the scale in the traces are: 20 *mV*/Div (*Y*-axis) and 5 *mS*/Div (*X*- axis). For panel (b), the scale in the traces are: 100 *mV*/Div (*Y*-axis) and 5 *mS*/Div (*X*- axis).

As indicated by the numerical simulations in Fig 4, the amplitude of the forcing has to be in an optimal moderate range to obtain logic operations. Figs 7 and 8 verify this behavior in electronic circuits. When the forcing amplitude is too small, the system tends to get stuck in some region of phase space and is unable to hop to the appropriate attractor. On the other hand, too large forcing amplitude results in the system hopping randomly from one sector of phase space to another, due to underlying double scroll attractors. Clearly, the intermediate forcing amplitude yields consistent logic operation, with appropriate attractor hopping induced only by changes in the input signal.

### Influence of noise: Generalized logical stochastic resonance

Lastly, we will investigate the effect of noise on the logic responses of the system [13, 14]. The first issue is to ascertain the robustness of the logic response with respect to ambient noise, i.e. we will check if noise degrades performance, or not. Secondly, we would like to investigate if there are some regions of dynamical behavior where noise aids the reliability of obtaining the correct logic output. In earlier studies it has been shown that a bistable system supporting two fixed point states, driven by a stream of sub-threshold input signals, yields enhanced probability of correct logic responses, in a window of optimal noise. This phenomena has been called *Logical Stochastic Resonance* (LSR) [15–20], and it has been realized in systems ranging from nanomechanical oscillators [21], coulomb-coupled quantum dots [22] and optical systems [23, 24] to chemical systems [25] and synthetic genetic networks [26–32]. Extensions of the idea to include the effect of periodic forcing was demonstrated in [33], where the width of the optimal noise window was shown to increase by utilizing periodic forcing, i.e. noise in conjunction with a periodic drive yielded consistent logic outputs for all noise strengths below a certain threshold. Further, the LSR concept has been demonstrated in coupled systems and higher-dimensional systems, with multiple steady states [26, 34, 35]. Specifically, two complementary gate operations were achieved simultaneously in a two-dimensional model of a gene network [26], indicating the flexible parallel processing potential of a biological system. In another direction for two coupled systems it was demostrated that, even when the individual systems receive only one logic input each, due to the interplay of coupling, nonlinearity and noise, they cooperatively respond to give a logic output that is a function of both inputs [35]. Further, the idea was extended to multi-stable dynamical systems with more than two stable fixed points, allowing one to obtain XOR logic, in addition to the AND (NAND) and OR (NOR) logic observed earlier [34].

Now in all its variations (some of which are detailed above), the concept of Logical Stochastic Resonace has so far been restricted only to *steady states*. In this work we explore the scope of the idea of LSR for the case of more complex attractors such as periodic cycles, or even *chaotic* attractors. Our basic question is then as follows: does the idea of Logical Stochastic Resonance extend beyond fixed point states, to more complex dynamical attractors? If it does indeed hold for more complex dynamics, we will have attained a *generalized* concept of Logical Stochastic Resonance.

**Fig 9:**
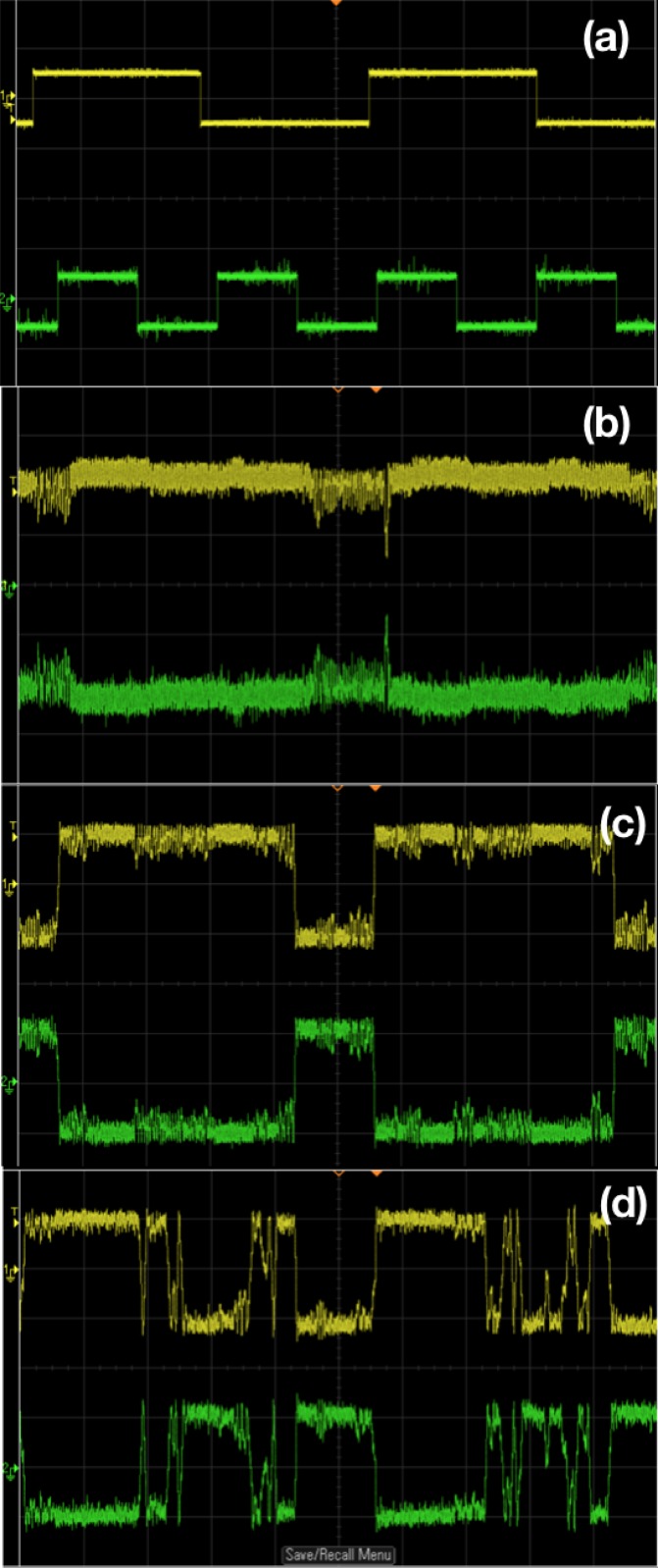
Realization of the OR/NOR logic gate at intermediate noise strengths in electronic circuit experiments. Panel (a) shows stream of inputs *I*_1_ and *I*_2_ (which take value −10 *mV* when logic input is 0 and value 10 *mV* when logic input is 1). Panels (b) to (d) show the output voltage *V*(*t*) (yellow) and *V*_*L*_(*t*) (green) for different noise strengths *D*: (i) 100 *mV*, (ii) 1.0 *V* and (iii) 1.5 *V*. Clearly panel (c) depicts logical OR output (with *V*(*t*) > 0 being logic output 1, and *V*(*t*) < 0 being logic output 0). The output voltage *V*_*L*_(*t*) (green) yields the complementary NOR logic gate response. The amplitude *A* of the sinusoidal signal is 100 *mV* and frequency is 8890 *Hz*. The bias voltage *b*, is fixed as 10 *mV*. For panel (a), the scale in the traces are: 20 *mV*/Div (*Y*-axis) and 5 *mS*/Div (*X*-axis). For panel (b-d), the scale in the traces are: 100 *mV*/Div (*Y*-axis) and 5 *mS*/Div (*X*-axis). For panel (a), the scale in the traces are: 20 *mV*/Div (*Y*-axis) and 5 *mS*/Div (*X*-axis). For panel (b-d), the scale in the traces are: 100 *mV*/Div (*Y*-axis) and 5 *mS*/Div (*X*-axis).

Fig 9 shows representative experimental results of this, for the system in Eq 1 under additive zero-mean Gaussian noise, given as:

$$\begin{array}{ccc} \dot{x} & = & y-g(x),\\ \dot{y} & = & -ay-x+b+I+f(t)+D\eta (t), \end{array}$$ (3)

where *η*(*t*) is a zero-mean Gaussian noise with variance 1, and parameter *D* gives the noise strength. In the circuit implementation displayed in Fig 5, *g*(*t*) now corresponds to *b* + *I* + *f*(*t*) + *Dη*(*t*).

**Fig 10:**
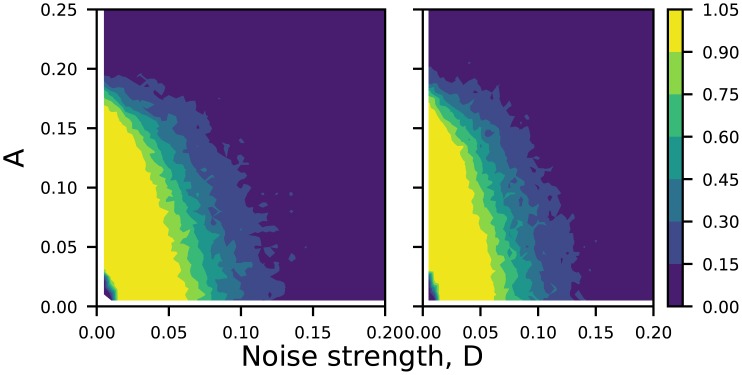
Probability of obtaining NOR logic, obtained from numerical simulations (with *b* = 0.025, on the left) and NAND logic (with *b* = −0.025, on the right) in the parameter space of forcing amplitude *A* (*y*-axis) and noise strength (*x*-axis) in Eq 3, with *ω* = 0.75 and inputs *I*_1_/*I*_2_ take value −0.025 when logic input is 0 and value 0.025 when logic input is 1.

Now, the forcing amplitude in the case illustrated is too small to yield appropriate attractor hopping that may be mapped to the output desired for logic operations, for subthreshold input signals. Naturally, when noise strength is too large, the system jumps randomly between attractors, and thus the system cannot yield any reliable output. When noise is zero or too small the system is essentially stuck in one dynamical attractor. However, remarkably, robust logic operations are realized when there is some noise in the system. So in the presence of moderate noise the system jumps from attractor to attractor in response to inputs consistently. Since these attractors are more complex than fixed points considered in earlier studies, these results offer a significant generalization of the concept of Logical Stochastic Resonance. The quantification of the reliability of obtaining a logic output through Logical Stochastic Resonance is depicted in Fig 10. It is clear that in relatively wide windows of moderate noise, the system yields logic operations with near certain probability i.e., *P*(*logic*)∼1. *Remarkably, note that the amplitude of the logic input signal is very low here, and may even be smaller than the noise strength*. For instance, in the particular illustrative example displayed in Fig 9, the input signal (*I* = 10*mV*) is 100 times smaller than the typical experimental noise strength in the optimal window of noise (∼1*V*).

**Fig 11:**
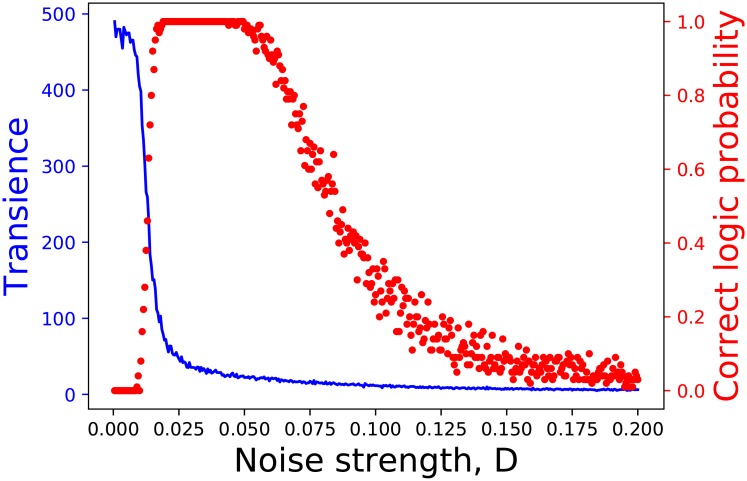
Transience (averaged over a random stream of inputs) as a function of noise strength. Here transience is estimated from numerical simulations, and is the time taken to reach the barrier from a well, when the input switches necessitate a change in the output. It is shown in terms of the scaled time in Eq 3, where 1 unit is 0.0000134 *sec*. The system parameters are those given in Fig 10.

We also observed the reduction of latency with increasing noise. This is evident in Fig 11. Clearly, the system responds to inputs more rapidly when noise intensity is higher. So the desired hopping between wells happens faster under the influence of stronger noise. This is yet another feature where noise assists performance.

### Generalized logical stochastic resonance with input-modulated parameters

**Fig 12:**
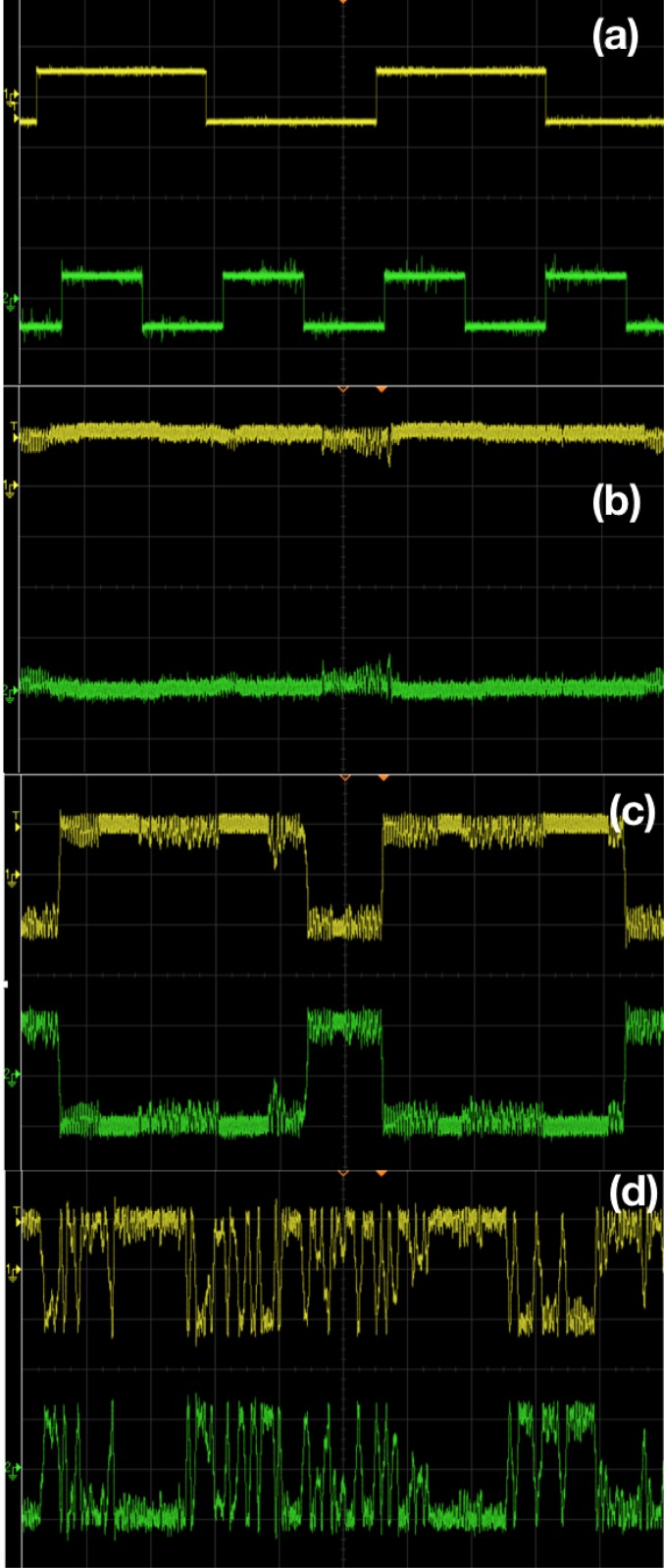
Generalized Logical Stochastic Resonance with Input-Modulated Parameters. Results from electronic circuit experiments, with panel (a) showing stream of inputs *I*_1_ and *I*_2_ (which take value −0.5 *V* when logic input is 0 and value 0.5 *V* when logic input is 1). Panels (b) to (d) show the output voltage *V*(*t*) (yellow) and *V*_*L*_(*t*) (green) for different noise strengths *D*: (i) 100 *mV*, (ii) 1.0 *V* and (iii) 1.5 *V*. Clearly panel (c) depicts logical OR output (with *V*(*t*) > 0 being logic output 1, and *V*(*t*) < 0 being logic output 0). The output voltage *V*_*L*_(*t*) (green) yields the complementary NOR logic gate response. The amplitude A of the sinusoidal signal is 100 *mV* and frequency is 8890 *Hz*. The bias voltage *b*, is fixed as 20 *mV*, and is modulated by the input signal streams as: *b*(*t*) = *b*(*I*_1_ + *I*_2_). A separate multiplier chip AD633 is used for modulation.

We demonstrate a further generalization of Logical Stochastic Resonance using input-modulated parameters, offering multiplicative perturbations to the system. Fig 12 shows representative experimental results of this, for the system in Eq 1 with the input signal *I* = *I*_1_ + *I*_2_ modulating parameter *b*:

$$\begin{array}{ccc} \dot{x} & = & y-g(x),\\ \dot{y} & = & -ay-x+b\left(I_{1}+I_{2}\right)+f\left(t\right)+D\eta \left(t\right), \end{array}$$ (4)

where *η*(*t*) is a zero-mean Gaussian noise with variance 1, and parameter *D* gives the noise strength. In the circuit implementation (cf. Fig 5), *g*(*t*) now corresponds to *b*(*I*_1_ + *I*_2_) + *f*(*t*) + *Dη*(*t*). The important distinction with the system in Eq 3 is that the *stream of inputs now modulate a parameter*. It is clearly evident that the system yields the appropriate logic output, as the input sets change, with the system switching as desired between different dynamical attractors bounded in distinct regions of phase space. Again, the logic output is obtained consistently in a window of moderate noise. This suggests that the scope of Logical Stochastic Resonance may be expanded to inputs-modulated parameters as well.

## Conclusion

The potential significance of the results obtained in this work are two-fold. The first is the proposal to implement fundamental logic operations by exploiting the switching between chaotic attractors. The underlying idea here is as follows: certain nonlinear systems can hop between dynamical attractors occupying different regions of phase space, under variation of parameters or initial states. We exploit this feature to obtain reliable logic operations by explicitly demonstrating the implementation of the fundamental NOR gate. The logic response can be morphed from NOR to NAND by a small change in the bias parameter, and this flexibility lays the foundation for general purpose reconfigurable circuitry [3, 36]. Further this system offers the advantage that very low-amplitude inputs (of the order of 10^−3^) yield highly amplified outputs (of the order of 1). The underlying reason for this is that small changes in the system yield significantly different dynamical outcomes. Additionally, different dynamical variables in the system yield complementary logic operations in parallel.

The second signficant result here is a generalization of the concept of Logical Stochastic Resonance. We show how the idea of LSR, which has so far been realized using steady states, may be extended to more complex dynamical attractors. So the noise floor can aid the reliability of the logic operations even when the logic operation is based on switching between states more complex than fixed points, such as hopping between periodic cycles or chaotic attractors. We also demonstrated that the generalized Logical Stochastic Resonance holds true for input-modulated parameters and multiplicative perturbations to the system.

In summary, we have shown how hopping between dynamical attractors of different geometries can be exploited for the implementation of logic gates. The ideas presented here, combining the research directions of Chaos Computing and Logical Stochastic Resonance, has potential to be realized in wide-ranging systems, and represents a new direction in exploiting chaotic systems to design computational devices.
